# Editorial: Discovery and function research of novel natural products

**DOI:** 10.3389/fchem.2026.1856543

**Published:** 2026-05-15

**Authors:** Peiran Liao, Peng Chen, Dan Xu, Fengwei Xie

**Affiliations:** 1 Guangzhou Comprehensive Experimental Station of National Medicinal Materials Industry Technology System, School of Chinese Materia Medica, Guangdong Pharmaceutical University, Guangzhou, China; 2 Chemistry and Chemical Engineering, Tarim University, Aral, China; 3 College of Food Science and Engineering, Guangdong Ocean University, Yangjiang, China; 4 China-Hungary Belt and Road Joint Laboratory on Food Science, Chongqing Key Laboratory of Specialty Food Co-Built by Sichuan and Chongqing, College of Food Science, Southwest University, Chongqing, China

**Keywords:** bioactivity, natural product analysis, natural products, pharmacology, synthesis, therapeutic discovery

Natural products have long been a valuable source of therapeutic agents, offering unparalleled chemical diversity and unique molecular scaffolds that continue to shape modern drug discovery. From anti-inflammatory and antioxidant to anti-tumor and metabolic disease treatments, nature-derived compounds remain indispensable. Despite centuries of exploration, the chemical space of medicinal plants, microorganisms, and marine organisms remains largely unexplored. There is an urgent need to isolate novel secondary metabolites, elucidate their structures, develop sustainable production platforms, and characterize their pharmacological mechanisms. This Research Topic was designed to capture recent advances in these interconnected areas, with a central focus on natural products as drivers of therapeutic innovation.

The collection includes five articles, comprising two comprehensive reviews and three original research papers, that collectively highlight the enduring value of natural products in drug discovery.

The reviews cover natural products derived from traditional medicinal herbs. In the first review, “The chemical structure, pharmacological activity, and clinical progress of Gentianae Radix et Rhizoma”, Liu et al. systematically catalogued the 172 natural constituents of Gentianae Radix et Rhizoma (Gentiana species), including iridoids (e.g., gentiopicroside and swertiamarin), triterpenoids, flavonoids, and alkaloids. The authors elucidated multi-target mechanisms involving NF-κB/MAPK pathways for anti-inflammation, Nrf2 activation for hepatoprotection, and Bax/Bcl-2 modulation for apoptosis induction. Importantly, the review integrated the clinical applications of the natural product gentiopicroside in treating herpes zoster, non-alcoholic fatty liver disease, and metabolic disorders, providing a comprehensive chemico-pharmacological-clinical framework for this genus. The second review, by Yang et al., “Advances in the structural characterization and pharmacological activity of *Salvia miltiorrhiza* polysaccharides”, focused on natural polysaccharides from *S. miltiorrhiza*, summarizing extraction methods, monosaccharide composition, and biological activities including immune regulation, anti-tumor activity, antioxidant activity, myocardial protection, and hepatoprotection. The authors emphasized the low toxicity and high research value of these natural products for nutraceutical and pharmaceutical development.

Natural products derived from plant cell culture are explored as a sustainable production platform. In an original research article, “Elicitor-driven enhancement of phenolic compounds in geranium callus cultures: phytochemical profiling via LC-MS/MS and biological activities”, Elbouzidi et al. investigated the elicitor-driven enhancement of natural phenolic compounds in *Pelargonium graveolens* (geranium) callus cultures. Treatment with chitosan and salicylic acid significantly increased the accumulation of total phenolic and flavonoid content. Profiling via LC-MS/MS analysis revealed marked increases in the natural products kaempferol and rutin. Furthermore, SA-elicited cultures demonstrated superior anti-tyrosinase and anti-elastase activities, highlighting the potential of this elicitation approach for developing natural anti-aging skincare products.

The quality control of natural products through spectrum-effect relationships was also investigated. In “Establishment of fingerprint of phenolic compounds in Semen *Ziziphi Spinosae* and study on the spectrum-effect relationship based on different preceding cropping areas”, Jiang et al. established HPLC fingerprint profiles of natural phenolic compounds in Semen *Ziziphi Spinosae* (SZS) from different preceding cropping areas. Seventeen common peaks were identified, including the natural products gallic acid, catechin, spinosin, and scutellarin. Multivariate analysis categorized 22 samples into three groups, and correlation analyses identified spinosin as a key contributor to total antioxidant activity. This study provides a practical reference for linking natural product chemical fingerprints to biological function in quality evaluation.

A novel mechanism from a natural product derivative was the focus of Yang et al. “Stemona alkaloid derivative induces ferroptosis of colorectal cancer cells by mediating carnitine palmitoyltransferase 1”. The authors explored the mechanism of a natural product-derived alkaloid derivative (SA-11) from Stemona species in colorectal cancer cells. Using cell metabolomics, the authors discovered that SA-11 induces ferroptosis via acylcarnitine accumulation, mediated by the upregulation of carnitine palmitoyltransferase 1 (CPT-1) without affecting CPT-2 levels. Exogenous acylcarnitine was found to enhance SA-11-induced ferroptosis, correlating with increased ROS production. This study is the first to report a natural product-inspired small molecule that induces ferroptosis through the regulation of acylcarnitine metabolism, offering a new therapeutic strategy for cancer treatment.

Collectively, these five articles illustrate the breadth of current natural product research, which encompasses everything from traditional medicinal herbs to plant cell culture systems and natural product derivatives. These studies address core themes such as anti-inflammatory, antioxidant, anti-aging, anti-tumor, and metabolic disease pharmacology, reaffirming that natural products remain an invaluable resource for therapeutic discovery. We thank all the authors, reviewers, and the *Frontiers in Chemistry* editorial team. We hope this collection inspires continued innovation in the discovery, synthesis, and pharmacological development of nature-derived therapeutics.

